# Implementation outcomes and strategies for depression interventions in low- and middle-income countries: a systematic review

**DOI:** 10.1017/gmh.2020.1

**Published:** 2020-03-02

**Authors:** Bradley H. Wagenaar, Wilson H. Hammett, Courtney Jackson, Dana L. Atkins, Jennifer M. Belus, Christopher G. Kemp

**Affiliations:** 1 Department of Global Health, University of Washington, Seattle, WA, USA; 2 Department of Epidemiology, University of Washington, Seattle, WA, USA; 3 Department of Psychology, University of Maryland, College Park, MD, USA

**Keywords:** Depression interventions, Implementation outcomes, Implementation science, Implementation strategies, Low- and middle-income countries

## Abstract

**Background:**

We systematically reviewed implementation research targeting depression interventions in low- and middle-income countries (LMICs) to assess gaps in methodological coverage.

**Methods:**

PubMed, CINAHL, PsycINFO, and EMBASE were searched for evaluations of depression interventions in LMICs reporting at least one implementation outcome published through March 2019.

**Results:**

A total of 8714 studies were screened, 759 were assessed for eligibility, and 79 studies met inclusion criteria. Common implementation outcomes reported were acceptability (*n* = 50; 63.3%), feasibility (*n* = 28; 35.4%), and fidelity (*n* = 18; 22.8%). Only four studies (5.1%) reported adoption or penetration, and three (3.8%) reported sustainability. The Sub-Saharan Africa region (*n* = 29; 36.7%) had the most studies. The majority of studies (*n* = 59; 74.7%) reported outcomes for a depression intervention implemented in pilot researcher-controlled settings. Studies commonly focused on Hybrid Type-1 effectiveness-implementation designs (*n* = 53; 67.1), followed by Hybrid Type-3 (*n* = 16; 20.3%). Only 21 studies (26.6%) tested an implementation strategy, with the most common being revising professional roles (*n* = 10; 47.6%). The most common intervention modality was individual psychotherapy (*n* = 30; 38.0%). Common study designs were mixed methods (*n* = 27; 34.2%), quasi-experimental uncontrolled pre-post (*n* = 17; 21.5%), and individual randomized trials (*n* = 16; 20.3).

**Conclusions:**

Existing research has focused on early-stage implementation outcomes. Most studies have utilized Hybrid Type-1 designs, with the primary aim to test intervention effectiveness delivered in researcher-controlled settings. Future research should focus on testing and optimizing implementation strategies to promote scale-up of evidence-based depression interventions in routine care. These studies should use high-quality pragmatic designs and focus on later-stage implementation outcomes such as cost, penetration, and sustainability.

## Introduction

For adults globally, mental, neurologic, and substance-use (MNS) disorders are the greatest contributor to years lived with disability (YLDs) – accounting for almost one-third of all YLDs [Institute for Health Metrics and Evaluation (IHME), 2017]. This finding is true in both high-income and low- and middle-income countries (LMICs). Depression alone accounts for 35% of all YLDs for mental disorders in countries with a low socio-demographic index, and over 6% of YLDs from any health condition (IHME, 2017). Yet, even with this widespread recognition of MNS disorders – and depression in particular – as key drivers of global disability, the gap between knowledge of evidence-based prevention and treatment approaches in the literature and its application in community settings is large. In high-income settings, only one in five patients with depression receive minimally-adequate treatment, with gaps increasing to one in nine in upper-middle-income countries and 1 in 27 for lower-middle-income countries (Thornicroft *et al*., 2017). Others have written that a comprehensive ‘mental health care gap’ would likely be much larger, as it would include the biomedical treatment gap, combined with the psychosocial care gap as well as the physical health care gap (Pathare *et al*., 2018). The recent landmark *Lancet Commission on Global Mental Health and Sustainable Development* (Patel *et al*., 2018) highlighted that even amongst high-income countries that have increased access to, and use of, evidence-based treatments for mood disorders from 1990 to 2015, the population-level prevalence of these conditions has not decreased. In fact, from 1991 to 2016, the disability burden of MNS disorders has steadily increased across both low- and high-income countries, although the largest increases (almost a doubling) have been seen in low-income countries (Patel *et al*., 2018).

To address the particularly large depression care gap in LMICs, the past decade has seen increased investment in pragmatic effectiveness trials to generate the evidence-base for mental health treatment in LMICs. The *Disease Control Priorities, 3rd Edition*, states that sufficient evidence exists for effectiveness and cost-effectiveness for preventative, drug, physical interventions, and psychosocial interventions for individuals with depressive disorders globally (Patel *et al*., 2016). Due to very limited trained mental health human resources in LMICs (Saxena *et al*., 2007; Kakuma *et al*., 2011), many interventions tested to date in LMICs have employed task-shifting, using lay health workers or peers to deliver low-intensity behavioral interventions, often in collaboration with primary care staff who can deliver psychopharmacological or other higher-intensity interventions as needed. In the past few years, this evidence base for depression treatment has matured, with numerous pragmatic effectiveness trials across LMICs showing effectiveness for trans-diagnostic delivered psychological therapies (Bolton *et al*., 2014; Murray *et al*., 2014; Weiss *et al*., 2015; Rahman *et al*., 2016; Bryant *et al*., 2017; Bonilla-Escobar *et al*., 2018; Murray *et al*., 2018; Khan *et al*., 2019), problem solving therapy (Chibanda *et al*., 2016*b*), interpersonal psychotherapy (Bolton *et al*., 2003, 2007; Bass *et al*., 2006), behavioral activation (Chowdhary *et al*., 2016; Patel *et al*., 2017; Weobong *et al*., 2017), cognitive behavioral therapy (Rahman *et al*., 2008; Maselko *et al*., 2015), cognitive processing therapy (Bass *et al*., 2013), family-based interventions (Jordans *et al*., 2013; Betancourt *et al*., 2014, 2017), and stepped-care multi-component interventions (Araya *et al*., 2003; Rojas *et al*., 2007; Patel *et al*., 2010, 2011; Adewuya *et al*., 2018; Jordans *et al*., 2019), among others. However, of these effective interventions, few have moved beyond the pilot phase of researcher-controlled implementation in LMICs to routine implementation at scale and with quality (Wainberg *et al*., 2017).

Almost a decade ago, four of the five highest-priority *Grand Challenges in Global Health* highlighted by Collins *et al*. (2011) focused on improving the implementation of existing treatments and expanding access to care – hallmarks of the field of implementation science. Recently, there have been calls by researchers and funders alike to increase the focus of the field of global mental health on implementation science, given the rapidly maturing evidence-base for effective treatments in LMICs (Betancourt and Chambers, 2016). The field of implementation science focuses on developing, testing, and utilizing implementation strategies to optimize the delivery of evidence-based interventions in routine practice (Eccles and Mittman, 2006). Implementation science is highly interdisciplinary, leveraging methods across traditional clinical research, the social sciences, public health, economics, political science, industrial engineering, and business to develop, test, and employ implementation strategies – methods to enhance implementation outcomes for evidence-based interventions in routine practice (Proctor *et al*., 2013). Recently, the field has adopted specific recommendations for specifying implementation strategies (Proctor *et al*., 2013), and Powell *et al*. (2015) have developed an initial compilation of 73 distinct implementation strategies through the Expert Recommendations for Implementing Change (ERIC) project (Powell *et al*., 2015). The success of an implementation strategy is most often measured through improvements in the implementation outcomes of acceptability, adoption, appropriateness, cost, feasibility, fidelity, penetration, and sustainability targeting a given evidence-based intervention (Proctor *et al*., 2011). Given the large burden of mental disorders in LMICs and the persistent care and treatment gaps, the field of implementation science has a critical role to play as evidence-based interventions are scaled-up and optimized for delivery across LMICs.

The aim of the current study was to systematically review the existing studies focused on implementation science for depression interventions in LMICs. Given the evolving and multi-disciplinary nature of the field of implementation science, the specific terms used define it are heterogeneous and mixed. Thus, the current review included all studies conducted in an LMIC that reported an implementation outcome (Proctor *et al*., 2011) tied to an intervention [a program, practice, principle, procedure, product, pill, or policy (Brown *et al*., 2017)] addressing depression as at least one of the primary outcomes of interest. Given the preeminence of implementation strategies to the field of implementation science, we also abstracted implementation strategy information and coded according to ERIC classifications (Powell *et al*., 2015). We chose to focus the current review on implementation science for interventions addressing depression given it represents the largest individual burden of MNS conditions in LMICs. We hope that this paper can provide a summary of the state of implementation research for depression interventions in LMICs, and aid stakeholders in identifying gaps and prioritizing future work in this area.

## Methods

### Protocol, registration, and reporting guidelines

This project is registered in the PROSPERO international prospective register of systematic reviews under record ID CRD42018084203 and title ‘Implementation science for depression interventions in low- and middle-income countries: a systematic review’. We followed the Preferred Reporting Items for Systematic Reviews and Meta-Analyses (PRISMA) reporting guidelines for systematic reviews (Liberati *et al*., 2009), which is available in online Supplementary Appendix 1.

### Search strategy

The lead author (BHW) searched four electronic bibliographic databases (PubMed; PsycINFO; CINAHL; and EMBASE) for articles published through 20 March 2019. We searched for articles including all four general search concepts, including: (1) depression; (2) an intervention, program, impact, or implementation; (3) implementation outcomes as defined by Proctor *et al*. (2011); and (4) studies conducted in LMICs, as defined by the World Bank Country and Lending Groups (2018). We developed a list of terms for each concept in collaboration with an information scientist. The PsycINFO search excluded dissertations, while the CINAHL focused only on scholarly peer-reviewed journals. See online Supplementary Appendix 2 for the detailed search strategy used.

### Study selection

Studies were included that: (1) were published in English; (2) were based in an LMIC according to the World Bank at the time of study data collection (including low-income; lower-middle-income; and upper-middle-income economies) (World Bank Country and Lending Groups, 2018); and (3) reported an implementation outcome as defined by Proctor *et al*. (2011) tied to an intervention [a program, practice, principle, procedure, product, pill, or policy (Brown *et al*., 2017)] targeting depression as at least one of the primary outcomes of interest (see Table 1 for specific implementation outcome definitions used). Unpublished and non-peer-reviewed research studies were excluded. We utilized Covidence to import bibliographic data and screen/review studies (Covidence Systematic Review Software, 2020). Two independent reviewers from a team of five (BHW; WHH; CJ; DLA; and JMB) independently reviewed each abstract at the title/abstract, full-text review, and the extraction phase. Studies passed the title/abstract phase if depression was mentioned and it was possible that the study had been conducted in an LMIC. Disagreements were resolved through discussion until consensus was reached. Articles were excluded if the full-text was unavailable after consulting with an information scientist at the University of Washington.

**Table 1.** Implementation outcome definitions used for systematic review based on Proctor's implementation outcome framework (Proctor *et al*., 2011)

Implementation outcome	Narrative definition	Population reporting	Stage of implementation reported	Type of data reporting	Specific study inclusion criteria	Exclusion criteria
Acceptability	Satisfaction with various aspects of intervention or implementation strategy	Consumer; Implementer	Pre-implementation; During implementation; Post-implementation	Qualitative; Quantitative	Must report formal analyses of quantitative or qualitative results of satisfaction with specific depression intervention or implementation strategy being tested	Excluded if general service satisfaction not linked to specific depression intervention or implementation strategy; Excluded if descriptive without formal analyses of client or consumer perceptions
Adoption	Initial implementation, utilization, or intention to try intervention or implementation strategy	Implementer; Organization; Setting	Pre-implementation; During implementation	Quantitative	Must be able to calculate numerator and denominator (no. of implementers, organizations, or settings adopting or intending to adopt/no. of eligible)	Excluded if qualitative results on use or intent to use not allowing calculation of proportion adopting
Appropriateness	Perceived fit, relevance, compatibility, usefulness, or practicability of intervention or implementation strategy	Consumer; Implementer; Organization; Setting	Pre-implementation	Qualitative; Quantitative	Must report formal qualitative or quantitative analyses of pre-implementation perceived fit of specific depression intervention or implementation strategy being tested	Excluded if general service appropriateness not focused on intervention or strategy of interest; Excluded if descriptive without formal analyses of client or consumer perceptions
Feasibility	Actual fit, relevance, compatibility, usefulness, or practicability of intervention or implementation strategy	Consumer; Implementer; Organization; Setting	During implementation; Post-implementation	Qualitative; Quantitative	Must report formal quantitative or qualitative analyses of post-implementation actual fit of specific depression intervention or implementation strategy being tested	Excluded if general service feasibility not focused on intervention or strategy of interest; Excluded if descriptive without formal analyses of client or consumer experiences
Fidelity	Degree to which an intervention or implementation strategy was implemented as intended	Implementer	During implementation	Quantitative	Must report formal analyses of fidelity to intervention or implementation strategy of interest; must be able to calculate numerator and denominator of (no. of implementers achieving fidelity/no. of implementing)	Excluded if general quality of care or delivery not focused on intervention or strategy of interest; Excluded if qualitative results on fidelity that do not allow calculation of proportion achieving fidelity
Cost	Cost of intervention or strategy delivery	Implementer; Organization; Setting	Post-implementation	Quantitative	Must report actual dollar amounts of implementation cost of intervention or implementation strategy of interest	Excluded if general costs of service delivery not tied to intervention or strategy of interest
Penetration	Degree of integration of intervention or implementation strategy within service setting and subsystems in non-controlled settings	Consumer; Implementer; Organization; Setting	During implementation; Post-implementation	Quantitative	Must be able to calculate numerator and denominator of either (no. of implementers, organizations, or settings delivering intervention or implementation strategy/no. of eligible) or (no. of consumers using a given intervention/no. of eligible); Depression intervention must be under routine institutional implementation, rather than controlled by researchers or external implementers	Excluded if results do not allow calculation of proportion utilizing intervention or implementation strategy with depression intervention delivered under routine conditions; Excluded if depression intervention under researcher-controlled implementation and not institutional implementation
Sustainability	Extent to which an intervention or implementation strategy is maintained within service setting's ongoing, stable operations	Organization; Setting	During implementation; Post-implementation	Qualitative; Quantitative	Defined as measurement of maintenance (either quantitative or qualitative) or ‘intent-to-continue use’ after initial pilot research-based funding has been rescinded and implementation is progressing under routine care conditions	Excluded if measurement only occurred during pilot research-based funding; Excluded if general sustainability not targeting intervention or implementation strategy of interest

### Data abstraction

Four authors (BHW; CJ; DLA; and JMB) independently piloted a structured abstraction form with five studies; all co-authors reviewed, critiqued, suggested improvements, and approved the final version. Two authors (BHW and WHH) independently abstracted study, intervention, and implementation strategy characteristics. After independently abstracting study information, the two authors (BHW and WHH) verified each abstraction, resolving any disagreement through discussion until consensus was reached. At the study level, we collected: (1) the year the study was published; (2) country and region of the study as defined by the World Bank in 2018 (World Bank Country and Lending Groups, 2018); (3) the primary research study design; (4) implementation outcomes reported; (5) detailed information on the depression intervention of interest; and (6) detailed information on implementation strategies tested (see Table 2 for categories of data abstraction).

**Table 2.** Study, depression intervention, implementation strategy, and implementation outcome descriptive statistics (*N* = 79)

Characteristic	*N* (%), unless noted
*Study characteristics*	
Published year, median (range)	2016 (2003–2019)
Region	
East Asia and Pacific	8 (10.1)
Europe and Central Asia	2 (2.5)
Latin America and Caribbean	13 (16.5)
Middle East and North Africa	3 (3.8)
South Asia	23 (29.1)
Sub-Saharan Africa	39 (36.7)
Primary research study design	
Mixed-methods	27 (34.2)
Qualitative	9 (11.4)
Quasi-experimental – controlled pre-post	3 (3.8)
Quasi-experimental – uncontrolled pre-post	17 (21.5)
Quasi-experimental – uncontrolled interrupted time-series	0 (0)
Quasi-experimental – controlled interrupted time-series	0 (0)
Quasi-experimental – regression discontinuity	0 (0)
Quasi-experimental – other	0 (0)
Randomized controlled trial – cluster	7 (8.9)
Randomized controlled trial – individual	16 (20.3)
Phase of implementation research^a^	
Pre-implementation assessment	4 (5.1)
Hybrid Type-1	53 (67.1)
Hybrid Type-2	0 (0.0)
Hybrid Type-3	16 (20.3)
T3	2 (2.5)
T4-1	4 (5.1)
T4-2	0 (0.0)
De-implementation (any phase)	0 (0.0)
*Implementation outcome characteristics*^b^	
Proctor^c^ implementation outcome reported	
Acceptability	50 (63.3)
Adoption	4 (5.1)
Appropriateness	14 (17.7)
Feasibility	28 (35.4)
Fidelity	18 (22.8)
Cost	14 (17.7)
Penetration	4 (5.1)
Sustainability	3 (3.8)
*Depression intervention characteristics*	
Stage of implementation of depression intervention	
Pilot researcher-controlled implementation	59 (74.7)
Delivered in routine care	20 (25.3)
Undergoing de-implementation	0 (0.0)
Implementation location of depression intervention	
Community	30 (38.0)
Health facility	47 (59.5)
Multi-level	2 (2.5)
Population implementing depression intervention	
Community members	7 (8.9)
Non-specialist healthcare workers	36 (45.6)
Nurses	6 (7.6)
Primary care physicians	8 (10.1)
Psychiatrists	2 (2.5)
Psychologists	3 (3.8)
Technology-based delivery	8 (10.1)
Multiple implementers	9 (11.4)
Modality of depression intervention	
Activity-based	3 (3.7)
Counseling	2 (2.5)
Education/information	1 (1.2)
Group counseling	1 (1.2)
Group psychotherapy	14 (17.3)
Individual psychotherapy	30 (38.0)
Medication	3 (3.7)
Multicomponent	27 (33.3)
*Implementation strategy characteristics*	
ERIC^d^ classification of implementation strategy	
Not testing implementation strategy – testing intervention	58 (73.4)
Conduct ongoing training	3 (3.8)
Create new clinical teams	1 (1.3)
Develop a formal implementation blueprint	1 (1.3)
Distribute educational materials	1 (1.3)
Facilitate relay of clinical data to providers	3 (3.8)
Increase demand	1 (1.3)
Provide clinical supervision	1 (1.3)
Revise professional roles	10 (12.7)
Implementation location of implementation strategy	
Not testing implementation strategy – testing intervention	58 (73.4)
Community	6 (7.6)
Health facility	14 (17.7)
District	1 (1.3)
Focal population utilizing implementation strategy	
Not testing implementation strategy – testing intervention	58 (73.4)
Community members	1 (1.3)
Non-specialist healthcare workers	11 (13.9)
Nurses	3 (3.8)
Primary care physicians	4 (5.1)
Policy makers	1 (1.3)
Multiple	1 (1.3)

a Phase of implementation research is defined as per Fig. 1.

b More than one implementation outcome possible, thus, total percentages exceed 100%.

c Implementation outcomes were defined as per Proctor's implementation outcome framework (Proctor *et al*., 2011).

d ERIC classification refers to the Expert Recommendations for Implementing Change project and the list of 73 distinct implementation strategies (Powell *et al*., 2015).

We defined implementation outcomes using the Proctor implementation outcomes framework (Proctor *et al*., 2011) (see Table 1). All implementation outcomes required actual measurement and data reporting from target populations; for example, qualitative narrative descriptions of overall ‘lessons learned’ without explicit data reporting were excluded.

We defined the phase of implementation research for each study across a modified translational research continuum (see Fig. 1). Building on consensus definitions from systematic reviews (Fort *et al*., 2017) and recent efforts to situate implementation research in the traditional translational research continuum (Brown *et al*., 2017), we envision depression interventions progressing from studies testing depression interventions for efficacy in highly-controlled settings (T2-1), to pragmatic intervention effectiveness trials with increased focus on external validity (T2-2). These studies come before those focused on testing and optimizing of implementation strategies for a given depression intervention (T3). This T3 phase has been titled ‘pure implementation research’ by Curran *et al*. (2012) and most often occurs after clinical intervention effectiveness has been shown. Hybrid effectiveness-implementation trials aim to speed the transition from pragmatic effectiveness trials (T2-2) to pure implementation research (T3) and the eventual work to evaluate and optimize the scale-up of evidence-based interventions for population health impact in T4-1. Following Curran *et al*. (2012), we defined Hybrid Type-1 as those studies with a primary aim of assessing intervention effectiveness and a secondary aim to report implementation outcomes for a depression intervention. Process evaluations published separately from main trial outcomes but embedded in larger Hybrid Type-1 studies were coded as Hybrid Type-1. Studies were coded as a ‘pre-implementation assessment’ if they reported implementation outcomes prior to implementation of a given depression intervention or strategy. In Fig. 1 we situate these studies as occurring before T2-2, as in our review they most commonly occurred before depression intervention effectiveness testing. Hybrid Type-2 studies were coded as having dual primary aims of assessing intervention effectiveness and testing an implementation strategy. Hybrid Type-3 studies were coded as having the primary aim of testing an implementation strategy while reporting on intervention effectiveness or patient-level outcomes. We define phase T4-1 as studies focused on evaluating and optimizing the scale-up of interventions and strategies in routine care for population health impact. We anticipate that these studies will often make use of known evidence-based implementation strategies found effective in stage T3. The last stage of the translational research continuum (T4-2) is the continuous optimization and improvement of ongoing routine delivery of an intervention that is being delivered at scale. This phase could follow models being proposed of ‘embedded research’ (Churruca *et al*., 2019; Lieu and Madvig, 2019) and/or ‘learning evaluation’ (Balasubramanian *et al*., 2015) where implementation researchers and practitioners collaborate to continuously improve the delivery of ongoing evidence-based interventions in routine systems. We consider de-implementation as occurring at each phase of the implementation research continuum. For example, while testing real-world intervention effectiveness (T2-2), one could also nest simultaneous study of de-implementation of other ineffective, wasteful, or harmful interventions targeting the same population (T2-2-DI).

**Fig. 1.**
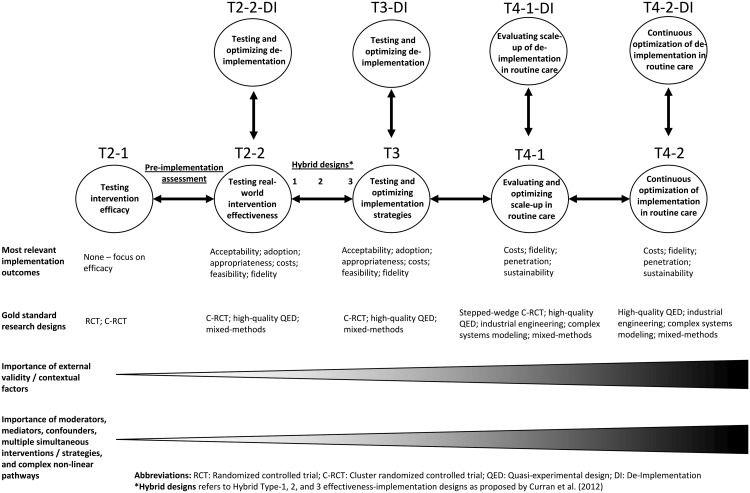
Situating implementation outcomes, research designs, and other key factors across the translational highway from efficacy research (T2-1) to continuous optimization of implementation in routine care (T4-2).

We defined the depression intervention as the specific proximal program, practice, principle, procedure, product, pill, or policy (Brown *et al*., 2017) that targeted depression symptoms. For depression intervention data abstraction, we coded the level of implementation as pilot researcher-controlled implementation, whether a given depression intervention was implemented in routine care, or whether the study was focused on de-implementation of a depression intervention. We also abstracted the implementation location of the depression intervention, the population implementing the intervention, and the modality of the depression intervention. Activity-based modalities were defined as depression interventions focused on a specific behavior for decreasing symptoms, such as running, yoga, or meditation. Counseling was defined as general one-on-one meetings not focused on a specific psychotherapy, such as general HIV or life-skills counseling. Education/information was defined as passive delivery of knowledge outside of a psychotherapy or counseling context, such as pamphlets or radio spots.

We coded implementation strategies only if the primary focus of the study was testing the effect of an implementation strategy, rather than testing the effects of a depression intervention. For studies meeting these criteria, we abstracted the implementation location of a given implementation strategy, the focal population utilizing the implementation strategy, and coded implementation strategy modalities according to the ERIC project's compilation of 73 distinct implementation strategies (Proctor *et al*., 2013; Powell *et al*., 2015).

### Analysis

We imported our final reporting excel sheet into Stata 15 for analyses. Analyses focused on generating a qualitative summary of research aims, methods, approaches, implementation outcomes, implementation strategies, and depression interventions tested to date to inform future research. Descriptively, we calculated percentages for categorical variables and for our continuous variable, year study was published, we calculated the median and range. Quantitative meta-analyses of study findings were not an aim of the current study given the heterogeneity in research questions, depression interventions, implementation strategies, and outcomes reported. See online Supplementary File S1 for full data abstraction form and study data.

## Results

### Study selection

**Fig. 2.**
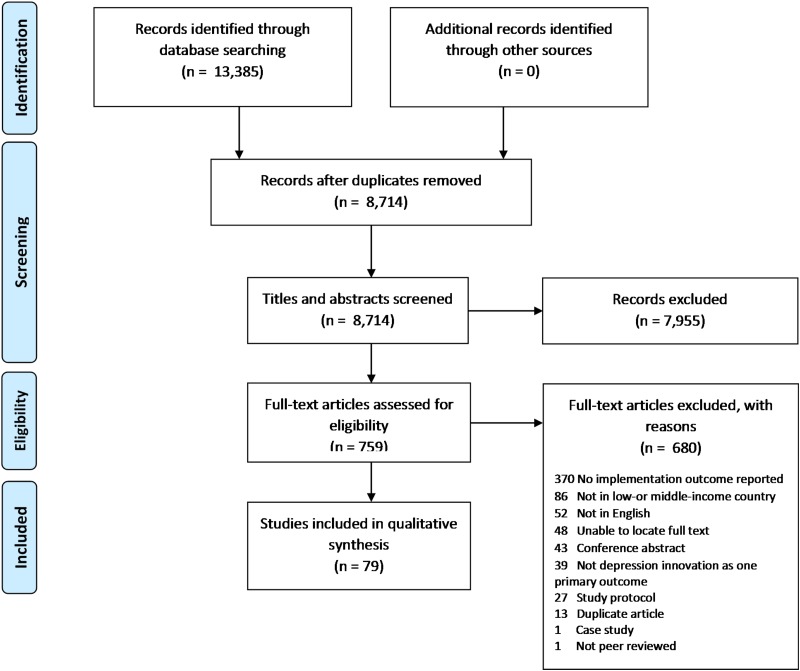
PRISMA flow diagram.

A total of 8714 unique studies were screened and 759 full-text articles were assessed for eligibility. Of these, 79 studies met our inclusion criteria (see Fig. 2 for PRISMA flow diagram). Of the 680 studies that were excluded at the full-text phase, the primary reason for exclusion was not reporting an implementation outcome (*n* = 370; 54.4%), not occurring in an LMIC (*n* = 86; 12.6%), not published in English (*n* = 52; 7.6%), and unable to locate the article full text (*n* = 48; 7.1%).

### Study characteristics

The 79 studies in our sample were published between 2003 and 2019, with the median published in 2016 and the mean being published in late 2014 (see Table 2). The number of studies has increased since 2015, with less than 10 studies published every year from 2003 to 2015, compared to 12 studies in 2016, 10 studies in 2017, 15 studies in 2018, and 8 studies published through 20 March 2019. The first three studies, published between 2003 and 2006, reported: (1) adoption and cost for the implementation strategy of ongoing training of primary health care nurses for depression treatment in Zimbabwe (Abas *et al*., 2003); (2) acceptability and fidelity of a psycho-educational depression intervention in Mexico (Lara *et al*., 2004); and (3) the costs associated with a multi-component stepped-care depression program for treating women with depression in Chile (Araya *et al*., 2006).

**Fig. 3.**
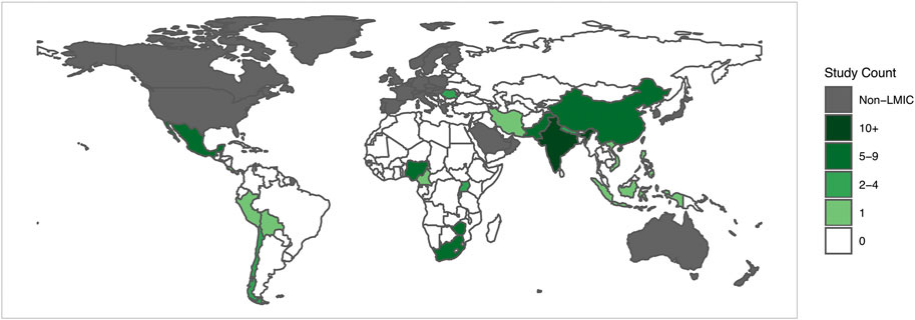
Thematic world map for distribution of included studies (*N* = 79).

The majority of studies were from Sub-Saharan Africa (*n* = 39; 36.7%), although only eight total countries in this region were represented, with South Africa (*n* = 9; 31.0%), Zimbabwe (*n* = 6; 20.7%), and Nigeria (*n* = 6; 20.7%) accounting for more than half of all articles published in the region (see Fig. 3 for world map of included studies). South Asia had the second greatest representation with 23 studies (29.1%); however, these were from only three countries: India (*n* = 14; 60.1%), Pakistan (*n* = 6; 26.1%), and Nepal (*n* = 3; 13.0%). Thirteen studies (16.5%) were from Latin America and the Caribbean, with the majority of studies coming from Mexico (*n* = 5; 38.5%) and Chile (*n* = 4; 30.8%). East Asia and the Pacific had relatively poor representation with only eight total studies (10.1%). Only five total studies were conducted across Europe and Central Asia (*n* = 2; 2.5%) and Middle East and North Africa (*n* = 3; 3.8%).

In terms of primary study designs, the most common design was mixed-methods (*n* = 27; 34.2%), using both qualitative and quantitative methods to address specific primary research questions. In terms of studies utilizing primary quantitative research study designs (*n* = 43; 54.4%), the most common design was quasi-experimental uncontrolled pre-post (*n* = 17; 21.5%), followed by individual randomized trials (*n* = 16, 20.3%), cluster randomized trials (*n* = 7; 8.9%), and quasi-experimental controlled pre-post designs (*n* = 3; 3.8%). No studies used quasi-experimental designs with the strongest causal inference, such as controlled interrupted time-series or regression discontinuity. Nine studies used pure qualitative research designs (11.4%).

In terms of phase of implementation research, the majority of studies were Hybrid Type-1 effectiveness-implementation designs (*n* = 53; 67.1), followed by Hybrid Type-3 designs (*n* = 16; 20.3), pre-implementation assessments (*n* = 4; 5.1%), T4-1 (*n* = 4; 5.1%), and T3 (*n* = 2; 2.5%). No studies assessed de-implementation at any phase, utilized Hybrid Type-2 designs, or targeted the last phase of T4-2. Three of the four studies targeting phase T4-1 were conducted in Chile (Vicente *et al*., 2007; Alvarado *et al*., 2012; Araya *et al*., 2012), with one study from Zimbabwe (Chibanda *et al*., 2016*a*). For example, Araya *et al*. (2012) conducted a mixed-methods study reporting on feasibility, cost, and sustainability to inform the optimization of implementation strategies for the National Depression Detection and Treatment Program which was in the process of scale-up across routine primary care in Chile. Similarly, Chibanda *et al*. (2016*a*) reported initial appropriateness and adoption of the ‘Friendship Bench’ program as it was in the process of being scaled-up across 60 primary care facilities in Zimbabwe.

### Implementation outcome characteristics

The 79 studies in our sample focused primarily on reporting early-stage implementation outcomes of acceptability (*n* = 50; 63.3%), feasibility (*n* = 28; 35.4%), and appropriateness (*n* = 14; 17.7%). Fidelity was also commonly measured (*n* = 19; 22.8%), as was cost (*n* = 14; 17.7%). Very few studies reported adoption (*n* = 4; 5.1%) or the later-stage implementation outcomes of penetration (*n* = 4, 5.1%) or sustainability (*n* = 3, 3.8%). In terms of studies reporting less-commonly reported implementation outcomes, Adewuya *et al*. (2018) reported adoption as the percentage of trained nurses (95.2%) who actively were delivering a pilot multicomponent screening, psychoeducation, psychological therapy, and medication intervention in primary care settings in Nigeria. Chatterjee *et al*. (2008) reported penetration as the percentage of patients (53%) who tested positive for a common mental disorder – including depression – who received the first session of psycho-education in their project testing the revision of professional roles (task-shifting) for a multicomponent depression intervention in routine primary care in India. Abas *et al*. (2016) reported on the quantitative sustainability of ‘Friendship Bench’ project activities up to 8 years after the initial pilot project ended and the depression intervention was formally integrated into routine care settings in Zimbabwe.

### Depression intervention characteristics

Seventy-five percent (*n* = 59) of depression interventions included in the studies occurred as part of pilot researcher-controlled implementation, rather than being implemented under routine care conditions. For example, in a Hybrid Type-1 study, Khan *et al*. (2019) reported the acceptability, feasibility, and initial clinical outcomes for patients attended by lay health workers randomized to implement group-based problem management plus (PM+) compared with enhanced usual care in Pakistan. Thus, the primary aim of this study was to test group PM+ as a novel depression intervention rather than testing an implementation strategy to enhance implementation outcomes for a depression intervention being delivered in routine implementation settings, as would occur in a Hybrid Type-3 or T3 study (see Table 3 for detailed study descriptions). By contrast, in a Hybrid Type-3 study, Shidhaye *et al*. (2017) reported the costs associated with an implementation strategy focused on increasing population-level demand for the existing routine depression care. The primary aim of their study was increase contact coverage of existing routinely-implemented depression interventions.

**Table 3.** Included studies (*N* = 79) and associated detailed study, intervention, and implementation strategy information

*N*	Author	Country	Research objectives	Description of primary depression intervention (implementing agent; where implemented; modality)	Description of primary implementation strategy (implementing agent; where implemented; ERIC classification)	Primary study design	Phase of implementation research	Depression intervention implementation stage	Implementation outcomes reported
1	Abas *et al*. (2003)	Zimbabwe	Explore barriers and facilitators to care of those with common mental disorders using routinely available data and face-to-face interviews with primary care staff, in public primary care clinics Harare	Multicomponent intervention delivered to community members by nurses at the facility level	Ongoing training conducted by nurses at the facility level	Mixed Methods	T3	Routine care	Adoption Cost
2	Abas *et al*. (2016)	Zimbabwe	Investigate acceptability and implementation of the ‘Friendship Bench Project’ using mixed-methods, 4-8 years after initial pilot study in Zimbabwe	Individual psychotherapy delivered to community members by non-specialist healthcare workers at the facility level	Primary focus is evaluating depression intervention	Mixed Methods	Hybrid Type-1	Routine care	Acceptability Feasibility Sustainability
3	Abas *et al*. (2018)	Zimbabwe	Pilot a task-shifted intervention to enhance adherence to HIV medication and improve depression outcomes in people living with HIV in Zimbabwe	Individual psychotherapy delivered to community members by non-specialist healthcare workers at the facility level	Primary focus is evaluating depression intervention	Individual-level Randomized Controlled Trial	Hybrid Type-1	Pilot researcher-controlled implementation	Acceptability Feasibility Fidelity
4	Abi Ramia *et al*. (2018)	Lebanon	Conduct bottom-up, community-driven qualitative cognitive interviewing from a multi-stakeholder perspective to inform the cultural adaptation of an Internet-delivered mental health intervention in Lebanon	Individual psychotherapy intervention delivered to community members through technology at the community level	Primary focus is evaluating depression intervention	Qualitative	Pre-implementation assessment	Pilot researcher-controlled implementation	Appropriateness
5	Adams *et al*. (2012)	Tanzania	Investigate feasibility of nurse-led antidepressant medication management of depression in an HIV clinic in Tanzania	Medication delivered to community members by nurses at the facility level	Nurses revising professional roles at the facility level	Quasi-Experimental – Uncontrolled Pre-Post	Hybrid Type-3	Routine care	Fidelity
6	Adewuya *et al*. (2018)	Nigeria	Develop and test the feasibility of a primary care worker-led psychological intervention as the main feature of a collaborative stepped care intervention for depression in Nigeria	Multicomponent intervention delivered to community members by multiple providers at the facility level	Primary focus is evaluating depression intervention	Mixed Methods	Hybrid Type-1	Pilot researcher-controlled implementation	Acceptability Adoption Appropriateness Feasibility Fidelity
7	Alampay *et al*. (2019)	Philippines	Examine the feasibility and acceptability of a local adaptation of a mindfulness-based cognitive therapy (MBCT) program for Filipino school children, facilitated by trained public school teachers	Group psychotherapy intervention delivered to community members by non-specialist healthcare workers at the community level	Primary focus is evaluating depression intervention	Individual-level Randomized Controlled Trial	Hybrid Type-1	Pilot researcher-controlled implementation	Acceptability Feasibility
8	Alvarado *et al*. (2012)	Chile	Evaluate a depression intervention implemented in the primary care setting in Chile	Multicomponent intervention delivered to community members by primary care physicians at the facility level	Primary care physicians revising professional roles at the facility level	Quasi-Experimental – Uncontrolled Pre-Post	T4-1	Routine care	Cost Sustainability
9	Andersen *et al*. (2016)	South Africa	Pilot a nurse-delivered cognitive behavioral therapy intervention (‘Ziphamandla’) to enhance adherence to HIV medication and improve depression in people living with HIV in South Africa	An individual psychotherapy intervention delivered to community members by nurses at the facility level	Primary focus is evaluating depression intervention	Quasi-Experimental – Uncontrolled Pre-Post	Hybrid Type-1	Pilot researcher-controlled implementation	Fidelity
10	Araya *et al*. (2006)	Chile	Evaluate the cost-effectiveness of a pilot depression treatment program for low-income women in the primary care setting in Santiago, Chile	A multicomponent intervention delivered to community members by non-specialist healthcare workers at the facility level	Primary focus is evaluating depression intervention	Individual-level Randomized Controlled Trial	Hybrid Type-1	Pilot researcher-controlled implementation	Cost
11	Araya *et al*. (2012)	Chile	Evaluate the scale-up of a depression treatment program in the primary care setting in Chile	A multicomponent intervention delivered to community members by primary care physicians at the facility level	Primary care physicians revising professional roles at the facility level	Mixed Methods	T4-1	Routine care	Feasibility Cost Sustainability
12	Asunción Lara *et al*. (2014)	Mexico	Describe a 4-year study monitoring the use of HDep, (‘Help for Depression’ or ADep, ‘Ayuda Para Depression’), an open access/free web-based, psycho-education, cognitive-behavioral intervention program in Mexico	An individual psychotherapy intervention delivered to community members by community members at the community level	Community members using mass media at the national level	Quasi-Experimental – Uncontrolled Pre-Post	Hybrid Type-3	Pilot researcher-controlled implementation	Acceptability
13	Lara *et al*. (2004)	Mexico	Investigate the degree of fidelity with which a psycho-educational intervention for women with depressive symptoms was delivered in Mexico	A group psychotherapy intervention delivered to community members by psychologists at the facility level	Primary focus is evaluating depression intervention	Qualitative	Hybrid Type-1	Pilot researcher-controlled implementation	Acceptability Fidelity
14	Atif *et al*. (2016)	Pakistan	Identify barriers and facilitators to delivering the ‘Barefoot Therapists’ maternal mental health intervention through peer volunteers in Pakistan	Individual psychotherapy intervention delivered to community members by non-specialist healthcare workers in the community setting	Primary focus is evaluating depression intervention	Qualitative	Hybrid Type-1	Pilot researcher-controlled implementation	Acceptability Feasibility
15	Atif *et al*. (2017)	Pakistan	Evaluate the adaptation and feasibility of the ‘Mother to Mother’ implementation of the therapy intervention ‘The Thinking Healthy Programme’ among mothers in India and Pakistan	An individual psychotherapy intervention delivered to community members by non-specialist healthcare workers in the community setting	Primary focus is evaluating depression intervention	Mixed Methods	Hybrid Type-1	Pilot researcher-controlled implementation	Acceptability Feasibility
16	Beardslee *et al*. (2011)	Costa Rica	Describe the adaptation of an evidence-based preventive depression-focused intervention for eventual widespread use in Costa Rica	A multicomponent intervention delivered to community members by non-specialist healthcare workers at the facility level	Primary focus is evaluating depression intervention	Qualitative	Hybrid Type-1	Pilot researcher-controlled implementation	Acceptability
17	Bella-Awusah *et al*. (2016)	Nigeria	Determine the effectiveness of a school-based cognitive behavioral therapy program (CBT) on adolescents with depression in southwestern Nigeria	A group psychotherapy intervention delivered to community members by psychiatrists at the community level	Primary focus is evaluating depression intervention	Cluster Randomized Controlled Trial	Hybrid Type-1	Pilot researcher-controlled implementation	Acceptability
18	Betancourt *et al*. (2014)	Rwanda	Assess the feasibility and acceptability of an intervention to reduce mental health problems and bolster resilience among children in households affected by caregiver HIV in Rwanda	A multicomponent intervention delivered to community members by psychologists at the community level	Primary focus is evaluating depression intervention	Mixed Methods	Hybrid Type-1	Pilot researcher-controlled implementation	Acceptability Fidelity
19	Betancourt *et al*. (2017)	Rwanda	Pilot the ‘Family Strengthening Intervention’, a family home-visit intervention designed to promote mental health and improve parent-child relationships in families with caregivers living with HIV in Rwanda	A multicomponent intervention delivered to community members by non-specialist healthcare workers at the community level	Primary focus is evaluating depression intervention	Individual-level Randomized Controlled Trial	Hybrid Type-1	Pilot researcher-controlled implementation	Acceptability
20	Burton *et al*. (2016)	Romania	Conduct a pilot RCT of Help4Mood, an interactive system with an embodied virtual agent (avatar) to assist in self-monitoring of patients receiving treatment for depression in Romania, Spain, and Scotland and the UK and evaluate the system use and acceptability of the pilot	A multicomponent intervention delivered to community members by technology at the community level	Primary focus is evaluating depression intervention	Cluster Randomized Controlled Trial	Hybrid Type-1	Pilot researcher-controlled implementation	Acceptability
21	Buttorff *et al*. (2012)	India	To carry out an economic evaluation of a task-shifting intervention for the treatment of depressive and anxiety disorders in primary-care settings in Goa, India	A multicomponent intervention delivered to community members by non-specialist healthcare workers at the facility level	Non-specialist healthcare workers revising professional roles at the facility level	Randomized Controlled Trial	Hybrid Type-3	Routine care	Cost Penetration
22	Chatterjee *et al*. (2008)	India	Integrate the MANAS intervention, an evidence-based treatment for common mental disorders, into routine primary care in Goa, India	A multicomponent intervention delivered to community members by non-specialist healthcare workers at the facility level	Non-specialist healthcare workers revising professional roles at the facility level	Mixed Methods	Hybrid Type-3	Routine care	Acceptability Appropriateness Feasibility Penetration
23	Chibanda *et al*. (2011)	Zimbabwe	Pilot a task-shifting primary mental health care intervention in a population with a high prevalence of people living with HIV in Zimbabwe	An individual psychotherapy intervention delivered to community members by non-specialist healthcare workers at the facility level	Primary focus is evaluating depression intervention	Mixed Methods	Hybrid Type-1	Pilot researcher-controlled implementation	Feasibility
24	Chibanda *et al*. (2016*a*)	Zimbabwe	Scale-up a depression intervention delivered by lay health workers in primary care facilities in Zimbabwe	An individual psychotherapy intervention delivered to community members by non-specialist healthcare workers at the facility level	Primary focus is evaluating depression intervention	Mixed Methods	T4-1	Routine care	Appropriateness Adoption
25	Chowdhary *et al*. (2016)	India	Evaluate The Healthy Activity Program, a lay counselor-delivered treatment for severe depression in India	An individual psychotherapy intervention delivered to community members by non-specialist healthcare workers at the facility level	Primary focus is evaluating depression intervention	Mixed Methods	Hybrid Type-1	Pilot researcher-controlled implementation	Acceptability Appropriateness Feasibility
26	Diez-Canseco *et al*. (2018)	Peru	Design, develop, and test a strategy to promote early detection, optimize referral, and access to treatment of patients with mental disorders attending public primary health care services in Lima, Peru	A multicomponent intervention delivered to community members by primary care physicians at the facility level	Primary care physicians revising professional roles at the facility level	Mixed Methods	Hybrid Type-3	Routine care	Appropriateness Feasibility Penetration
27	Doumit *et al*. (2018)	Lebanon	Assess the feasibility, acceptability, and preliminary effects of a cognitive-behavioral intervention [Creating Opportunities for Patient Empowerment (COPE)] on depression, anxiety, and quality of life (QOL) in a sample of adolescent refugees in Lebanon	A group psychotherapy intervention delivered to community members by multiple providers at the community level	Primary focus is evaluating depression intervention	Quasi-Experimental – Uncontrolled Pre-Post	Hybrid Type-1	Pilot researcher-controlled implementation	Acceptability
28	Duffy *et al*. (2017)	Zimbabwe	Pilot a nurse-led integration of mental health and HIV services in Zimbabwe	A multicomponent intervention delivered to community members by nurses at the facility level	Non-specialist healthcare workers revising professional roles at the facility level	Mixed Methods	Hybrid Type-3	Pilot researcher-controlled implementation	Acceptability Feasibility
29	Dwommoh *et al*. (2018)	South Africa	Investigate the cost-effectiveness of a brief motivational interviewing (MI) intervention *v.* a combined intervention of MI and problem-solving therapy (MI-PST) for reducing substance use and depression among patients presenting to emergency departments in South Africa	An individual psychotherapy intervention delivered to community members by non-specialist healthcare workers at the facility level	Primary focus is evaluating depression intervention	Individual-level Randomized Controlled Trial	Hybrid Type-1	Pilot researcher-controlled implementation	Cost
30	Fisher *et al*. (2014)	Vietnam	Adapt and field-test the Thinking Healthy Program (THP) for perinatal depression and anxiety treatment in Vietnam	A group psychotherapy intervention delivered to community members by non-specialist healthcare workers at the community level	Primary focus is evaluating depression intervention	Mixed Methods	Hybrid Type-1	Pilot researcher-controlled implementation	Acceptability Appropriateness
31	Fuhr *et al*. (2019)	India	Assess the effectiveness and cost-effectiveness of the Thinking Healthy Programme (THP) when peer-delivered in Goa, India	An individual psychotherapy intervention delivered to community members by community members at the community level	Primary focus is evaluating depression intervention	Individual-level Randomized Controlled Trial	Hybrid Type-1	Pilot researcher-controlled implementation	Cost
32	Gallegos *et al*. (2012)	Mexico	Evaluate the effectiveness of the FRIENDS for Life program, a social and emotional skills program implemented in an orphanage in Mexico	A group psychotherapy intervention delivered to community members by community members at the community level	Primary focus is evaluating depression intervention	Quasi-Experimental – Uncontrolled Pre-Post	Hybrid Type-1	Pilot researcher-controlled implementation	Acceptability
33	Guo *et al*. (2018)	China	Implement an mHealth intervention program for people living with HIV in China via the popular social media app WeChat	An education/information intervention delivered to community members through technology at the community level	Primary focus is evaluating depression intervention	Individual-level Randomized Controlled Trial	Hybrid Type-1	Pilot researcher-controlled implementation	Acceptability
34	Gureje *et al*. (2015)	Nigeria	Pilot a program integrating mental health into primary care in Osun State, Nigeria	A multicomponent intervention delivered to community members by multiple providers at the facility level	Multiple providers conducting ongoing training at the facility level	Quasi-Experimental – Uncontrolled Pre-Post	T3	Routine care	Fidelity
35	Gureje *et al*. (2019)	Nigeria	Compare high-intensity treatment (HIT) with low-intensity treatment (LIT) for perinatal depression in Nigeria	An individual psychotherapy intervention delivered to community members by nurses at the facility level	Primary focus is evaluating depression intervention	Cluster Randomized Controlled Trial	Hybrid Type-1	Pilot researcher-controlled implementation	Cost
36	Hashemi *et al*. (2012)	Iran	Compare the efficacy of nortriptyline with that of fluoxetine in the treatment of patients with major depressive disorder in Iran	A medication intervention delivered to community members by primary care physicians at the facility level	Primary focus is evaluating depression intervention	Individual-level Randomized Control Trial	Hybrid Type-1	Routine care	Acceptability
37	Isa *et al*. (2018)	Nigeria	Investigate the effects of a psychological intervention that includes psycho-education and basic elements of cognitive behavioral therapy (CBT) on medication-treated adolescents with depression in Nigeria	A group psychotherapy intervention delivered to community members by psychiatrists at the facility level	Primary focus is evaluating depression intervention	Quasi-Experimental – Uncontrolled Pre-Post	Hybrid Type-1	Pilot researcher-controlled implementation	Acceptability
38	Janevic *et al*. (2016)	Bolivia	Evaluate the feasibility of an automated telephonic interactive voice response (IVR) depression self-care service among Bolivian primary care patients	A counseling intervention delivered to community members through technology at the community level	Primary focus is evaluating depression intervention	Quasi-Experimental – Uncontrolled Pre-Post	Hybrid Type-1	Pilot researcher-controlled implementation	Acceptability
39	Jordans *et al*. (2013)	Burundi	Evaluate the impact of a brief parenting psychoeducation intervention on children's mental health in Burundi	A group psychotherapy intervention delivered to community members by non-specialist healthcare workers at the community level	Primary focus is evaluating depression intervention	Quasi-Experimental – Controlled Pre-Post	Hybrid Type-1	Pilot researcher-controlled implementation	Acceptability
40	Jordans *et al*. (2019)	Nepal	Evaluate the impact of a district mental healthcare plan for depression, psychosis, alcohol use disorder, and epilepsy as part of the Programme for Improving Mental Health Care (PRIME) in Chitwan District, Nepal	A multicomponent intervention delivered to community members by multiple providers at the multiple levels	Policy-makers developing a formal implementation blueprint at the district level	Quasi-Experimental – Uncontrolled Pre-Post	Hybrid Type-3	Pilot researcher-controlled implementation	Fidelity Penetration
41	Khan *et al*. (2019)	Pakistan	Evaluate the feasibility and acceptability of the locally adapted Group Problem Management Plus (PM+) intervention for women in the conflict-affected settings in Swat, Pakistan	A group psychotherapy intervention delivered to community members by non-specialist healthcare workers at the community level	Primary focus is evaluating depression intervention	Cluster Randomized Controlled Trial	Hybrid Type-1	Pilot researcher-controlled implementation	Acceptability Feasibility
42	Maulik *et al*. (2016)	India	Implement an approach incorporating mobile-based electronic decision support systems (EDSS) to provide services for common mental disorders, combined with a community-based anti-stigma campaign, in Andhra Pradesh, India	A multicomponent intervention delivered to community members by primary care physicians at the facility level	Non-specialist healthcare workers at the community level facilitating the relay of clinical data to providers at the facility level	Mixed Methods	Hybrid Type-3	Routine care	Acceptability Feasibility
43	McIntyre *et al*. (2018)	South Africa	Examine the application of Mindfulness Based Stress Reduction (MBSR) for HIV-infected individuals in South Africa	An activity-based intervention delivered to community members by primary care physicians at the community level	Primary focus is evaluating depression intervention	Mixed Methods	Hybrid Type-1	Pilot researcher-controlled implementation	Acceptability
44	Mehrotra *et al*. (2018)	India	Develop and pilot-test PUSH-D (Practice and Use Self-Help for Depression), a self-help intervention for depression, in an urban setting in India	An individual psychotherapy intervention delivered to community members by technology at the community level	Primary focus is evaluating depression intervention	Quasi-Experimental – Uncontrolled Pre-Post	Hybrid Type-1	Pilot researcher-controlled implementation	Acceptability
45	Munodawafa *et al*. (2017)	South Africa	Explore the lay counselor experience of delivering a task-shared psycho-social intervention for perinatal depression in Khayelitsha, South Africa	An individual psychotherapy intervention delivered to community members by non-specialist healthcare workers at multiple levels	Primary focus is evaluating depression intervention	Qualitative	Hybrid Type-1	Pilot researcher-controlled implementation	Feasibility Fidelity
46	Murray *et al*. (2014)	Multiple (Iraq; Thailand)	Describe the Common Elements Treatment Approach (CETA) for adults presenting with mood or anxiety problems developed specifically for use with lay counselors in low- and middle-income countries	An individual psychotherapy intervention delivered to community members by community members at the facility level	Primary focus is evaluating depression intervention	Mixed Methods	Hybrid Type-1	Pilot researcher-controlled implementation	Adoption Fidelity
47	Myers *et al*. (2019)	South Africa	Examine the feasibility and acceptability of integrating into chronic disease care two approaches to community health worker-delivered mental health counseling in South Africa	An individual psychotherapy intervention delivered to community members by non-specialist healthcare workers at the facility level	Primary focus is evaluating depression intervention	Quasi-Experimental – Uncontrolled Pre-Post	Hybrid Type-1	Pilot researcher-controlled implementation	Feasibility
48	Nakimuli-Mpungu *et al*. (2014)	Uganda	Assess the feasibility, acceptability and impact on depression, functioning, social support and self-esteem of a manualized culturally sensitive group support psychotherapeutic intervention for depressed HIV-affected Ugandan adults	A group psychotherapy intervention delivered to community members by non-specialist healthcare workers at the community level	Primary focus is evaluating depression intervention	Quasi-Experimental – Controlled Pre-Post	Hybrid Type-1	Pilot researcher-controlled implementation	Acceptability
49	Nakimuli-Mpungu *et al*. (2017)	Uganda	Evaluate the effectiveness of a group support psychotherapy for depression treatment among people with HIV/AIDS in northern Uganda	A group psychotherapy intervention delivered to community members by non-specialist healthcare workers at the community level	Primary focus is evaluating depression intervention	Mixed Methods	Hybrid Type-1	Pilot researcher-controlled implementation	Acceptability Feasibility Fidelity
50	Naveen *et al*. (2013)	India	Develop and pilot a yoga therapy module for patients with depression in India	An activity-based intervention delivered to community members by community members at the community level	Primary focus is evaluating depression intervention	Quasi-Experimental – Uncontrolled Pre-Post	Hybrid Type-1	Pilot researcher-controlled implementation	Acceptability Appropriateness
51	Oladeji *et al*. (2015)	Nigeria	Pilot a stepped care intervention package for depression in the primary care setting in Nigeria	A multi-component intervention delivered to community members by non-specialist healthcare workers at the facility level	Non-specialist healthcare workers revising professional roles at the facility level	Cluster Randomized Controlled Trial	Hybrid Type-3	Routine care	Appropriateness Feasibility Fidelity
52	Patel *et al*. (2017)	India	Pilot The Healthy Activity Program (HAP), a lay counselor-delivered brief psychological treatment for severe depression, in primary care in India	An individual psychotherapy intervention delivered to community members by non-specialist healthcare workers at the facility level	Primary focus is evaluating depression intervention	Individual-level Randomized Controlled Trial	Hybrid Type-1	Pilot researcher-controlled implementation	Fidelity Cost
53	Pence *et al*. (2014)	Cameroon	Adapt measurement-based care for depressed HIV-infected patients in Cameroon and completed a pilot study to assess feasibility, safety, acceptability, and preliminary efficacy	A multicomponent intervention delivered to community members by non-specialist healthcare workers at the facility level	Non-specialist healthcare workers creating new clinical terms at the facility level	Quasi-Experimental – Uncontrolled Pre-Post	Hybrid Type-3	Routine care	Acceptability Fidelity
54	Petersen *et al*. (2014)	South Africa	Assess the feasibility of a group-based counseling intervention for HIV-positive patients with depression in primary health care in South Africa using a task shifting approach	A group psychotherapy intervention delivered to community members by non-specialist healthcare workers at the facility level	Primary focus is evaluating depression intervention	Individual-level Randomized Controlled Trial	Hybrid Type-1	Pilot researcher-controlled implementation	Acceptability Feasibility
55	Rahman, (2007)	Pakistan	Identify challenges and opportunities in developing a psychological intervention for perinatal depression in rural Pakistan	An individual psychotherapy intervention delivered to community members by non-specialist healthcare workers at the community level	Primary focus is evaluating depression intervention	Mixed Methods	Pre-implementation assessment	Pilot researcher-controlled implementation	Acceptability Appropriateness
56	Ramaiya *et al*. (2018)	Nepal	Implement a dialectical behavioral therapy intervention among women with history of suicidality and evaluate its feasibility and acceptability	A group psychotherapy intervention delivered to community members by non-specialist healthcare workers at the facility level	Primary focus is evaluating depression intervention	Mixed Methods	Hybrid Type-1	Pilot researcher-controlled implementation	Acceptability Feasibility
57	Sava *et al*. (2009)	Romania	Assess the cost-effectiveness and cost-utility of cognitive therapy (CT), rational emotive behavioral therapy (REBT), and fluoxetine (Prozac) for major depressive disorder (MDD) in Romania	A multicomponent intervention delivered to community members by multiple providers at the facility level	Primary focus is evaluating depression intervention	Individual-level Randomized Controlled Trial	Hybrid Type-1	Pilot researcher-controlled implementation	Cost
58	Seedat *et al*. (2008)	South Africa	Pilot a consumer psychoeducation program to improve antidepressant adherence in South Africa	A medication intervention delivered to community members by multiple providers at the facility level	Community members distributing education materials at the community level	Quasi-Experimental – Uncontrolled Pre-Post	Hybrid Type-3	Routine care	Acceptability Feasibility
59	Shidhaye *et al*. (2017)	India	Assess whether implementation of the community mental health program VISHRAM was associated with an increase in the proportion of people with depression who sought treatment in India	A multicomponent intervention delivered to community members by multiple providers at the facility level	Non-specialist healthcare workers increasing demand at the community level	Quasi-Experimental – Uncontrolled Pre-Post	Hybrid Type-3	Routine care	Cost
60	Shinde *et al*. (2013)	India	Perform a qualitative analysis of the intervention experience of the MANAS trial, a lay counselor led collaborative stepped care intervention in Goa, India	A multicomponent intervention delivered to community members by multiple providers at the facility level	Non-specialist healthcare workers revising professional roles at the facility level	Qualitative	Hybrid Type-3	Routine care	Acceptability
61	Sikander *et al*. (2019)	Pakistan	Adapt the Thinking Healthy Programme (THP) for delivery by volunteer peers and assess its effectiveness and cost-effectiveness in Rawalpindi, Pakistan	An individual psychotherapy intervention delivered to community members by community members at the community level	Primary focus is evaluating depression intervention	Cluster Randomized Controlled Trial	Hybrid Type-1	Pilot researcher-controlled implementation	Cost
62	Singla *et al*. (2014)	India	Evaluate a peer-led quality assessment of psychological treatments in Goa, India	An individual psychotherapy intervention delivered to community members by non-specialist healthcare workers at the facility level	Non-specialist healthcare workers providing clinical supervision at the facility level	Mixed Methods	Hybrid Type-3	Pilot researcher-controlled implementation	Acceptability Fidelity
63	Smith Fawzi *et al*. (2012)	Haiti	Examine the feasibility and assess the preliminary effectiveness of a psychosocial support group intervention for HIV-affected youth and their caregivers in central Haiti	A group counseling intervention delivered to community members by non-specialist healthcare workers at the community level	Primary focus is evaluating depression intervention	Mixed Methods	Hybrid Type-1	Pilot researcher-controlled implementation	Acceptability
64	Sorsdahl *et al*. (2015)	South Africa	Evaluate feasibility and preliminary responses to a screening and brief intervention program for maternal mental disorders within the context of primary care in South Africa	An individual psychotherapy intervention delivered to community members by non-specialist healthcare workers at the facility level	Primary focus is evaluating depression intervention	Mixed Methods	Hybrid Type-1	Pilot researcher-controlled implementation	Feasibility
65	Sullivan *et al*. (2016)	Mexico	Describe the process of culturally adapting and disseminating the Community Advocacy Project to improve depression among survivors of intimate partner violence in Monterrey, Mexico	A counseling intervention delivered to community members by non-specialist healthcare workers at the community level	Primary focus is evaluating depression intervention	Mixed Methods	Hybrid Type-1	Pilot researcher-controlled implementation	Acceptability Feasibility
66	Surjaningrum *et al*. (2018)	Indonesia	Examine the feasibility of an integrated mental health task-sharing intervention focused on identifying perinatal depression in Surabaya, Indonesia	A multicomponent intervention delivered to community members by non-specialist healthcare workers at the facility level	Non-specialist healthcare workers revising professional roles at the community level	Qualitative	Pre-implementation assessment	Routine care	Appropriateness
67	Tang *et al*. (2015)	China	Explore the advantages and challenges of implementing a village doctor-based cognitive behavioral therapy intervention in treating late-life depression in rural China	An individual psychotherapy intervention delivered to community members by non-specialist healthcare workers at the community level	Primary focus is evaluating depression intervention	Qualitative	Hybrid Type-1	Pilot researcher-controlled implementation	Appropriateness Feasibility Fidelity
68	Tewari *et al*. (2017)	India	Implement a mental health services delivery model that leverages technology and task sharing to facilitate identification and treatment of common mental disorders in rural Andhra Pradesh, India	A multicomponent intervention delivered to community members by primary care physicians at the facility level	Non-specialist healthcare workers facilitating the relay of clinical data to providers at the community level	Mixed Methods	Hybrid Type-3	Routine care	Acceptability Feasibility Fidelity
69	Tiburcio *et al*. (2016)	Mexico	Develop and evaluate the usability of the web-based Help Program for Drug Abuse and Depression in Mexico	An individual psychotherapy intervention delivered to community members through technology at the community level	Primary focus is evaluating depression intervention	Qualitative	Pre-implementation assessment	Pilot researcher-controlled implementation	Acceptability Appropriateness Feasibility
70	Tomita *et al*. (2016)	South Africa	Assess the feasibility of SMS-based methods to screen for depression risk among refugees in South Africa and to compare its reliability and acceptability with face-to-face consultation	A multicomponent intervention delivered to community members by multiple providers at the facility level	Technology facilitating relay of clinical data to nurses at the community level	Quasi-Experimental – Uncontrolled Pre-Post	Hybrid Type-3	Pilot researcher-controlled implementation	Acceptability
71	Tripathy *et al*. (2010)	India	Assess the effects of participatory women's groups on birth outcomes and maternal depression in a largely tribal and rural population in three districts in eastern India	A multicomponent intervention delivered to community members by community members at the community level	Primary focus is evaluating depression intervention	Cluster Randomized Controlled Trial	Hybrid Type-1	Pilot researcher-controlled implementation	Cost
72	Vicente *et al*. (2007)	Chile	Evaluated the results of a brief 2-day educational training program for Chilean primary care physicians to improve diagnosis and treatment of depression	A multicomponent intervention delivered to community members by primary care physicians at the facility level	Primary care physicians conducting ongoing training at the facility level	Quasi-Experimental – Controlled Pre-Post	T4-1	Routine care	Acceptability
73	Walker *et al*. (2018)	Nepal	Assess the feasibility and acceptability of a psychosocial support package for people receiving treatment for multidrug-resistant TB in Nepal	A multicomponent intervention delivered to community members by non-specialist healthcare workers at the facility level	Primary focus is evaluating depression intervention	Mixed Methods	Hybrid Type-1	Pilot researcher-controlled implementation	Acceptability Feasibility
74	Watt *et al*. (2017)	Tanzania	Evaluate a psychological intervention for women receiving surgical care for obstetric fistula in Tanzania	An individual psychotherapy intervention delivered to community members by nurses at the facility level	Primary focus is evaluating depression intervention	Individual-level Randomized Controlled Trial	Hybrid Type-1	Pilot researcher-controlled implementation	Acceptability Fidelity
75	Weobong *et al*. (2017)	India	Evaluate the sustained effectiveness and cost-effectiveness of the Healthy Activity Programme, a brief psychological treatment for depression delivered by lay counselors in primary care in India	An individual psychotherapy intervention delivered to community members by non-specialist healthcare workers at the facility level	Primary focus is evaluating depression intervention	Individual-level Randomized Controlled Trial	Hybrid Type-1	Pilot researcher-controlled implementation	Cost
76	Yang *et al*. (2018)	China	Develop and test a culturally tailored, brief three-session CBT skills-based intervention for HIV+ men who have sex with men (MSM) integrated into primary care in China	An individual psychotherapy intervention delivered to community members by psychologists at the facility level	Primary focus is evaluating depression intervention	Mixed Methods	Hybrid Type-1	Pilot researcher-controlled implementation	Acceptability Feasibility
77	Yang *et al*. (2019)	China	Test the feasibility and acceptability of an 8-week online mindfulness intervention for pregnant women as an approach to reduce depressive and anxious symptoms in China	An individual psychotherapy intervention delivered to community members by technology at the facility level	Primary focus is evaluating depression intervention	Individual-level Randomized Controlled Trial	Hybrid Type-1	Pilot researcher-controlled implementation	Acceptability
78	Yeung *et al*. (2018)	China	Examine the feasibility, safety, and effectiveness of using an online computerized cognitive behavioral therapy (CBT) for treating patients with depression in China	An individual psychotherapy intervention delivered to community members by technology at the facility level	Primary focus is evaluating depression intervention	Individual-level Randomized Controlled Trial	Hybrid Type-1	Pilot researcher-controlled implementation	Acceptability
79	Zafar *et al*. (2014)	Pakistan	Develop and integrate a cognitive behavioral therapy-based maternal psychosocial well-being intervention (the five-pillars approach) into a child nutrition and development program in Pakistan	An individual psychotherapy intervention delivered to community members by non-specialist healthcare workers at the community level	Primary focus is evaluating depression intervention	Mixed Methods	Hybrid Type-1	Pilot researcher-controlled implementation	Acceptability Appropriateness Feasibility Fidelity

The depression interventions of interest were most often delivered at the health facility level (*n* = 47; 59.5), followed by the community level (*n* = 30; 38.0%), and multi-level programs (*n* = 2; 2.5%). Most depression interventions were delivered by non-specialist healthcare workers (*n* = 36; 45.6%) who did not have specific specialist mental health training and were not trained as a nurse or other clinical providers. Other common providers included team-based delivery (9, 11.4%); primary care physicians (8, 10.1%), and technology-based delivery (8, 10.1%). The modality for depression intervention was most commonly individual psychotherapy (*n* = 30, 38.0%), followed by multi-component interventions (*n* = 27; 33.3%) that commonly included both psychotherapy and psychotropic medication. Group psychotherapy was also common, with 14 studies (17.3%). Few studies focused on medication alone (*n* = 3; 3.7%), activity-based treatments (*n* = 3; 3.7%), or education/information (*n* = 1; 1.2%).

### Implementation strategy characteristics

Seventy-three percent of studies (*n* = 58) had the primary aim of testing the depression intervention of interest, rather than testing an implementation strategy to enhance implementation outcomes for a given depression intervention. Of the 21 studies (26.5%) testing an implementation strategy, the majority employed the strategy at the health facility level (*n* = 14; 66.6%), followed by the community (*n* = 6; 28.6%) and the district levels (*n* = 1; 4.8%). For example, in a Hybrid Type-3 study, Maulik *et al*. (2016) assessed the acceptability and feasibility of a community-based electronic decision-support implementation strategy to facilitate the relay of clinical data from non-specialist health workers in the community to primary care providers to improve routinely-provided public-sector depression care in India. Additionally, Jordans *et al*. (2019) reported fidelity and penetration resulting from an implementation strategy to work with policy-makers at the district level to develop a formal implementation blueprint focused on improving routinely-delivered depression care in Nepal.

Of the 21 studies testing implementation strategies, the majority employed non-specialist healthcare workers in strategy implementation (*n* = 11; 52%), followed by primary care physicians (*n* = 4; 19.0%) and nurses (*n* = 3; 14.2%). The ERIC classification of implementation strategies highlighted revising professional roles as the dominant strategy (*n* = 10; 47.6%). These studies primarily focused on testing task-sharing approaches to optimize implementation outcomes of depression interventions. For example, Alvarado *et al*. (2012) studied the cost and sustainability of revising the professional roles of primary care providers to implement a stepped-care multicomponent depression treatment program in routine care settings in Chile. Buttorff *et al*. (2012) reported the cost and penetration of testing the strategy of revising professional roles to have non-specialist workers deliver depression care in routine primary care settings in India.

Other implementation strategies commonly tested included facilitating the relay of clinical data to providers (*n* = 3; 14.3%) and conducting ongoing training (*n* = 3; 14.3%). For example, Tewari *et al*. (2017) reported the acceptability, feasibility, and fidelity of a community-based electronic decision-support implementation strategy to facilitate the relay of clinical depression data from the community to primary care providers in India. Gureje *et al*. (2015) reported fidelity associated with the conducting ongoing training of primary healthcare workers in the WHO Mental Health Gap Action Programme Intervention Guide to integrate depression treatment with primary care in Nigeria. Five of the eight distinct ERIC strategies tested were covered by only one study, including: (1) creating new clinical teams; (2) developing a formal implementation blueprint; (3) distributing educational materials; (4) increasing demand; and (5) providing clinical supervision. Sixty-five of the 73 distinct ERIC strategies were not represented by studies in our sample.

## Discussion

Our systematic review found a relatively large body of implementation research targeting depression interventions in LMICs. Researchers are increasing their use of modern methods in implementation science, with a growing focus on reporting implementation outcomes, testing implementation strategies, and using pragmatic study designs to optimize the delivery of evidence-based interventions in routine settings. Nevertheless, our review identified significant gaps in the literature. The vast majority of existing implementation research focuses on early-stage implementation outcomes, such as acceptability, appropriateness, and feasibility, with a paucity of studies focusing on later-stage outcomes such as cost, penetration, and sustainability. In addition, only one quarter of studies had the primary aim of testing an implementation strategy. The majority of studies reported implementation outcomes as part of a ‘process evaluation’ complementary to a pragmatic trial or pilot study focused on evaluating clinical effectiveness for a depression intervention. Thus, the bulk of studies identified in our review were labeled as ‘Hybrid Type-1 effectiveness-implementation’ designs (Curran *et al*., 2012). With this continued focus on clinical effectiveness, it is no surprise that three quarters of included studies were studying a depression intervention delivered under pilot researcher-controlled implementation, rather than in routine care. Furthermore, we found only four studies that were focused on evaluating and optimizing the scale-up of depression interventions for population health impact in LMICs. We also found specific LMIC regions with few existing studies, including the Middle East and North Africa and Europe and Central Asia. Overall, our findings corroborate a recent systematic review focused on implementation science for stigma reduction interventions in LMICs (Kemp *et al*., 2019*a*) which found that the majority of studies utilized Hybrid Type-1 designs, qualitative research methods, and reported the implementation outcomes of acceptability and feasibility.

Assessing depression intervention effectiveness in a randomized controlled trial along with a mixed-methods process evaluation to assess early-stage implementation outcomes tied to depression intervention acceptability, appropriateness, feasibility, or initial fidelity is an optimal approach for a Hybrid Type-1 research design. Yet, even among the 57 studies at this stage, only two (Murray *et al.*, 2014; Adewuya *et al*., 2018) reported the early-stage implementation outcome of adoption. This is not surprising since pilot researcher-controlled implementation projects often hire separate research staff as implementers and thus ‘non-participation’ (or lack of adoption) of these separate paid staff is not common. Nevertheless, the lack of reporting on adoption, paired with the dearth of studies reporting on penetration, means that little is known regarding the uptake, initial implementation, or potential routine institutionalization of depression interventions in LMICs – key implementation outcomes for effective and sustained scale-up of depression interventions. Future research, even at T2-2 and Hybrid Type-1 stages, could focus on approximating real-world routine implementation conditions to allow effective reporting of provider and organizational adoption and implementation costs. Where possible, these studies should also build in funding for assessment of later-stage penetration and sustainability (maintenance) once research-based implementation has ended as has been advocated by the RE-AIM evaluation framework for decades (Glasgow *et al*., 1999). Researchers working at this stage could also consider increased utilization of high-quality quasi-experimental designs with optimal design features for causal inference, rather than the current dominance of randomized trials and poor-quality uncontrolled pre-post quasi-experimental designs (Bärnighausen *et al*., 2017; Geldsetzer and Fawzi, 2017; Reeves *et al*., 2017). The highest-quality quasi-experimental designs for causal inference in routine implementation settings of controlled interrupted time-series (Bernal *et al*., 2017) and regression discontinuity (Bor *et al*., 2014) were not represented in our review.

Of the 21 studies testing implementation strategies, over 70% (*n* = 16) were Hybrid Type-3 designs, followed by studies at phases T3 (*n* = 2; 9.5%), and T4-1 (*n* = 3; 14.3%). Compared to Hybrid Type-1 studies, Hybrid Type-3 studies were more likely to use quasi-experimental designs (7 of 16; 43.8% *v*. 10 of 53; 18.9%) and less likely to use randomized designs (2 of 16; 12.5% *v*. 21 of 53; 39.6%). Furthermore, no study with a primary aim to test an implementation strategy used individual randomization, compared to 27.6% (17 of 58) of studies with a primary aim to test intervention effectiveness. This pattern is in line with the fact that pragmatic cluster randomized trials and high-quality quasi-experimental designs are the gold standard research designs for testing and optimizing implementation strategies for an intervention delivered in routine care. Using individual randomization at the later phases of the implementation research continuum is logistically difficult, and can potentially alter routine systems of implementation, decreasing both internal and external validity. As expected, implementation outcome distributions were similar across Hybrid Type-1 and Hybrid Type-3 studies. The six studies situated at T3 and T4-1 were more likely to focus on the later-stage implementation outcomes of cost (*n* = 3; 50.0%) and sustainability (*n* = 2; 33.3%); these studies were also more likely to report adoption (*n* = 2; 33.3%). As visualized in Fig. 1, we suggest that the most relevant implementation outcomes at the testing implementation strategy phase (T3) are similar at T2-2 or when using Hybrid designs. Although, at stage T3, implementation outcome reporting should target both the implementation strategy being tested along with the target depression intervention. For example, if testing an implementation strategy to improve the adoption of a depression intervention, researchers will also be interested in measuring, assessing, and optimizing the adoption of the implementation strategy.

Almost half of the 21 studies testing implementation strategies focused on assessing the effectiveness of revising professional roles, most often through task-sharing to provide depression care led by primary care providers or lay health workers in a stepped-care approach supervised by specialist providers. Only eight of the 73 distinct ERIC implementation strategies (Powell *et al*., 2015) had any representation in our review, with five strategies being tested in only one study. For efficient generation of evidence given the large number of potential distinct implementation strategies – not to mention multicomponent or blended groups of strategies – we suggest future implementation research considers increased use of adaptive trial designs, such as sequential multiple assignment randomized trials (SMART); (Lei *et al*., 2012) or multiphase optimization strategy trials (MOST) (Collins *et al*., 2007) which can allow testing multiple strategies across multiple doses in a single trial. Quasi-experimental designs, natural experiments, and descriptive analyses utilizing routine data systems and naturally-occurring heterogeneity in implementation strategies should also be prioritized to help rapidly identify, test, and optimize the scale-up of promising implementation strategies in LMICs (Wagenaar *et al*., 2016).

Overall, our findings that the majority of existing implementation studies on depression interventions in LMICs are utilizing Hybrid Type-1 designs could reflect three things. First, that researchers and implementers working in LMICs do not believe we have sufficient evidence for depression intervention effectiveness across diverse contexts to move to later implementation research phases. Second, few researchers and implementers have the necessary skills or knowledge to effectively extend past traditional clinical research designs focused on depression intervention effectiveness testing toward testing implementation strategies (T3), optimizing scale-up in routine systems settings (T4-1), or continuous optimization in routine systems settings (T4-2). Third, research funders have yet to fully embrace the field of implementation science and the value of moving beyond traditional clinical research paradigms.

To address the first point, we argue that with the publishing of the *Disease Control Priorities, 3rd Edition* in 2016 outlining that sufficient evidence exists for effectiveness and cost-effectiveness for depression interventions globally (Patel *et al*., 2016), the time is now for increased investments in implementation research and to move beyond testing intervention effectiveness and toward testing implementation strategies (T3), scale-up (T4-1), and continuous optimization of routine implementation (T4-2). The second point will require the development and re-training of a pipeline of researchers with expertise outside of traditional clinical research paradigms centered on highly-controlled randomized trials as the lone gold standard research design. As the field of global mental health progresses away from pragmatic clinical intervention studies and toward optimizing the scaling-up (T4-1) of best-evidence interventions and associated implementation strategies and continuous ‘embedded research’ and ‘learning evaluation’ of interventions and strategies already scaled-up (T4-1), we anticipate that a number of key evolutions will occur. Implementation outcome reporting will center on later stage costs, fidelity, penetration, and sustainability of both interventions and implementation strategies. Clinical researchers will have to be re-trained or partner with various disciplinary experts to implement analytical designs focused more on high-quality quasi-experimental approaches, continuous improvement and systems optimization borrowing from industrial engineering and business domains, along with novel systems modeling approaches (Royston, 2011; Sherr *et al*., 2014; Wagner *et al*., 2019). These methods will be of increasing importance due to the need to model, test, and continuously improve complex webs of multiple evidence-based interventions and implementation strategies for depression operating across contexts and in non-linear pathways (Galea *et al*., 2010; Kemp *et al*., 2019*b*). Studies operating at the level of entire districts, provinces, or nations in LMICs will have the necessary primary focus on external validity and will focus on continuous reporting of implementation outcomes using routine data systems (Victora *et al*., 2004; Wagenaar *et al*., 2016). To catalyze later phases of implementation research in LMICs, funding agencies, governments, and stakeholders will need to recognize their role in funding ongoing real-world implementation of depression interventions, not simply focusing on funding one-off research-based implementation projects.

This review has several important limitations. First, given available resources, we were only available to review papers published in English, likely systematically excluding studies emanating from certain LMICs. Second, this review focused on peer-reviewed studies. Given the barriers to publishing in international peer-reviewed journals, especially for LMIC investigators, this likely biases our findings to papers published by internationally-connected and funded investigators. Third, given the heterogeneous language and terminology used in the burgeoning field of implementation science, making strict distinctions between ‘evidence-based depression interventions’ and ‘implementation strategies’ of interest, as well as coding implementation strategies into distinct ERIC classifications (Powell *et al*., 2015) is challenging. Similarly, coding the stage of implementation research is also difficult and somewhat qualitative in nature, as implementation researchers themselves have not adopted a comprehensive and common language to situate their studies along the implementation research continuum. Fourth, given the size of this review, we focused data abstraction on implementation outcomes and high-level coding of depression intervention characteristics. We did not abstract detailed information on implementation context, the use of implementation science frameworks, or on the diversity of specific depression interventions – areas that could be of interest for future systematic reviews. Last, we did not code the level of analysis for implementation outcomes which we suggest future researchers conduct. This is important to distinguish, for example, client-level adoption or sustainability (reach and maintenance, respectively in the RE-AIM framework, Glasgow *et al*., 1999) from provider- or organizational-level adoption or sustainability.

## Conclusions

Our systematic review of implementation outcomes and strategies for depression interventions in LMICs found that existing research has focused largely on early-stage implementation outcomes. Most studies had the primary aim of testing the pragmatic effectiveness of a depression intervention in pilot researcher-controlled settings paired with a ‘process evaluation’ to collect information on implementation context. Thus, the majority of studies were Hybrid Type-1 effectiveness-implementation designs, with very few studies focused on evaluating and optimizing strategies for scale-up of depression interventions in routine care. Even though the ostensible focus of the field of implementation science is to test implementation strategies to optimize the real-world implementation of evidence-based interventions, only a quarter of studies had a primary aim to test implementation strategies for interventions implemented in routine care. Approximately half of these studies testing implementation strategies were focused on testing revised professional roles, or task-shifting, for depression intervention implementation. Only eight of the 73 distinct ERIC implementation strategies were represented in our systematic review. Future implementation research should focus on testing implementation strategies and optimizing the use of evidence-based strategies to scale-up and improve the quality of routine depression care. These studies should use high-quality pragmatic research designs such as controlled interrupted time-series, regression discontinuity, stepped-wedge randomized trials, and novel complex systems modeling approaches, as well as focus on later-stage implementation outcomes such as cost, penetration, and sustainability. Certain LMIC regions, such as Middle East and North Africa and Europe and Central Asia could be prioritized for investments given the paucity of existing studies.

## Supplementary material

For supplementary material accompanying this paper visit https://doi.org/10.1017/gmh.2020.1. click here to view supplementary material

## Data

All data are available in online Supplementary File S1.
